# Best practices for vascular arterial access and closure: a contemporary guide for the cardiac catheterization laboratory

**DOI:** 10.3389/fcvm.2024.1349480

**Published:** 2024-03-14

**Authors:** Moemen Eltelbany, Matteo Fabbri, Wayne B. Batchelor, Lindsey Cilia, Aaron Ducoffe, Kendall Endicott, Kelly Epps, Amika McBurnie, Richard Neville, Carolyn Rosner, Matthew W. Sherwood, David Spinosa, Alexander G. Truesdell, Cassandra Vorgang, Abdulla A. Damluji, Behnam N. Tehrani

**Affiliations:** ^1^ Inova Schar Heart and Vascular, Falls Church, VA, United States; ^2^ Virginia Heart, Falls Church, VA, United States; ^3^ Department of Cardiovascular Medicine, Johns Hopkins University, Baltimore, MD, United States

**Keywords:** vascular access and closure, major bleeding, major vascular complication, vacular closure device, large bore access

## Abstract

More than 1 million transcatheter-based cardiovascular procedures across the spectrum of interventional cardiology are performed annually in the United States. With the expanded indications for and increased complexities associated with these procedures, interventional cardiologists are expected to possess the requisite expertise to complete these interventions safely and effectively. While the art of vascular access and closure remains a prerequisite and critical skillset in contemporary practice, there remain significant variations in the techniques employed, resulting in the bleeding and vascular complications encountered in clinical practice. With an increasing recognition of the potential merits to standardized approaches to vascular access and closure, cardiovascular societies have put forth recommendations around best practices for performing these procedures in the cardiac catheterization laboratories. In this review, we aim to: (1) Examine the evolving definitions of bleeding and vascular complications; (2) Review best practices for transradial and transfemoral access and closure, including for large bore procedures; and (3) Highlight knowledge gaps and proposed areas of clinical research pertaining to vascular access which may inform clinical practice and potentially optimize the outcomes of patients undergoing transcatheter-based cardiac and vascular interventions.

## Introduction

**Figure 1:**
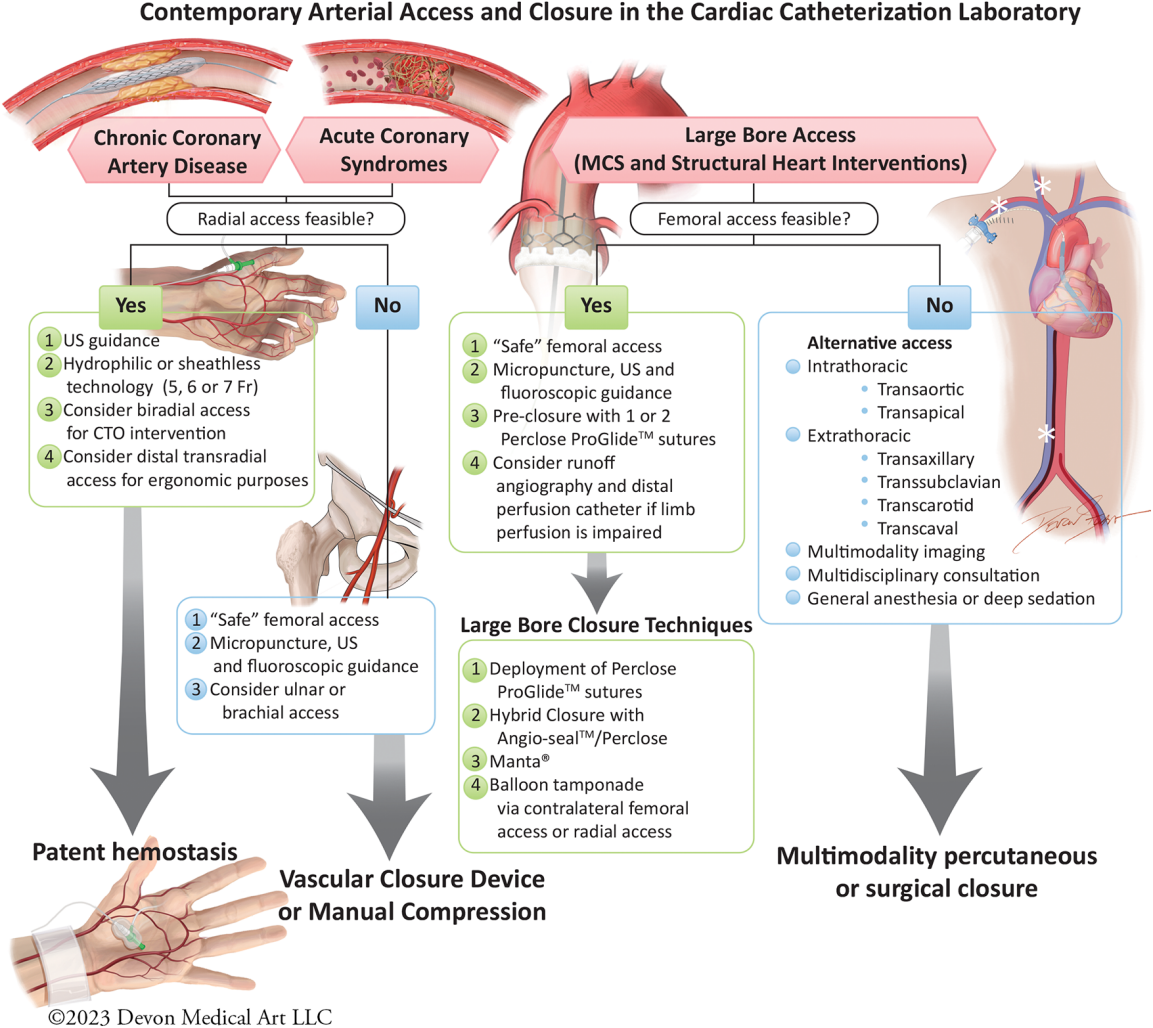
Contemporary arterial access and closure in the cardiac catheterization laboratory.

Cardiovascular disease is the leading cause of mortality in the United States, with a prevalence of nearly 50% in all adults over the age of 20, and greater than 2.2 million hospitalizations and 1,000 deaths occurring on a daily basis (1). The population is also increasingly aging with an enhanced burden of cardiac co-morbidities and associated frailty syndromes (2). As a result, more than one million transcatheter-based procedures are performed annually to diagnose and treat cardiac conditions (3). There have also been significant advances in the scope of technologies to perform transcatheter-based cardiac and vascular procedures, covering the broad spectrum of coronary, peripheral and structural heart disease. Patients are also increasingly requiring mechanical circulatory support (MCS) devices to treat cardiogenic shock (CS) and to facilitate high risk percutaneous coronary interventions (PCI) (4, 5). With the expanded indications for and increased complexities associated with cardiac catheterizations, interventional cardiologists are expected to possess the expertise required to perform these procedures safely and effectively. Despite these clinical demands, there remain significant variations in how these techniques are employed, thus contributing to the bleeding and vascular complications encountered in clinical practice (6, 7). As a result of increasing recognition of the potential merits to standardized approaches to vascular access and closure, cardiovascular societies have put forth recommendations around best practices for these techniques (3, 8). In this review, we aim to: (1) Examine the evolving definitions of bleeding and the clinical importance of this complication in daily practice; (2) Review best practices for transradial arterial access (TRA) for coronary angiography and PCI; (3) Assess contemporary techniques for standard and large-bore transfemoral arterial access (TFA) and closure (Figure 1); and (4) Highlight knowledge gaps and proposed areas of clinical research pertaining to vascular access which may potentially optimize the outcomes of patients undergoing transcatheter-based cardiac procedures.

### Major bleeding following transcatheter-based cardiac procedures

Historically, varying definitions of major bleeding have been studied across different PCI registries and clinical trials, and it has been shown to be a powerful predictor of morbidity and mortality (9–13). A summary of the most commonly used bleeding nomenclatures is highlighted in Table 1. While bleeding following cardiac catheterization may occur from a variety of sources, more than 50% of cases are due to access site complications (14, 15). An analysis of 15,968 patients from the GLOBAL LEADERS study, a contemporary randomized clinical trial comparing 1-month vs. 12-month dual antiplatelet therapy following PCI in an all-comer patient population, reported a 6.6% risk of Bleeding Academic Research Consortium (BARC) 2, 3, or 5 bleeding (16). Overall, 11 percent of patients with a bleeding event died during the two-year follow-up, and the presence of even a minor periprocedural bleed imparted a 1.8-fold increase-risk for mortality beyond 1 year (16). The clinical and economic impacts of periprocedural bleeding are significant, with up to 14% of patients undergoing a blood transfusion and each complication contributing to nearly $12,000 in increased costs (15–17). Notwithstanding the stabilizing effects of increased circulatory volume and oxygen carrying capacity, the practice of blood transfusion is associated with a deleterious impact on clinical outcomes following PCI, with 3-fold increased risk of mortality and major adverse cardiac events (18). This effect is likely multifactorial, due to a combination of increased platelet reactivity, upregulation of plasminogen activator inhibitor proteins, reductions in 2, 3-diphosphoglyceric acid and nitric oxide levels in the stored red blood cells (19–22).

**Table 1:** Major bleeding definitions in contemporary interventional cardiology.

GRACE (12)	GUSTO (13)	TIMI (10)	REPLACE-2 (9)	BARC (11)
**Major:** - Requiring a transfusion of ≥2 units PRBCs - Resulting in a decrease in hematocrit ≥ 10%. - Occurring intracerebrally - Resulting in stoke or death.	**Severe:** - Intracerebral hemorrhage, leading to hemodynamic instability, requiring treatment. **Moderate:** - Requiring blood transfusion, but not associated with hemodynamic instability. **Mild**: Bleeding that does not meet above criteria	**Non-CABG Related Bleeding** **Major:** - Any intracranial bleeding (excluding microhemorrhages <10 mm evident only on gradient-echo MRI), clinically overt signs of hemorrhage associated with a drop in hemoglobin of ≥5 g/dl, or fatal bleeding (bleeding that results in death within 7 days). **Minor:** bleeding with Hgb drop between 3 and 5 g/dl. **Requiring Medical Attention:** - Any overt sign of hemorrhage that meets one of the following criteria and does not meet criteria for a major or minor bleeding event, as defined above. - Requiring intervention (medical practitioner-guided medical or surgical treatment to stop or treat bleeding, including temporarily or permanently discontinuing or changing the dose of a medication or study drug). - Leading to or prolonging hospitalization. - Prompting evaluation (leading to an unscheduled visit to a healthcare professional and diagnostic testing; either laboratory or imaging. - Minimal: Any overt bleeding event that does not meet the criteria.	**Major:** Intracranial, intraocular, or retroperitoneal. Blood loss with Hgb drop of at least 3 g/dl. Transfusion ≥2 units of PRBC. **Minor:** - Bleeding not meeting major criteria.	**Type 1:** bleeding that is not actionable and doesn't require urgent studies, hospitalization, or treatment. **Type 2:** overt, actionable sign of hemorrhage, with at least one of the following: requiring neurosurgical, or medical intervention hospitalization, or increased level of care. **Type 3a:** overt bleeding plus a hemoglobin drop of 3–5 g/dl, any transfusion with overt bleeding. **Type 3b:** overt bleeding with a hemoglobin drop of 5 g/dl, Cardiac tamponade, Bleeding requiring surgical intervention, Bleeding requiring vasoactive agents. **Type 3c:** intracranial hemorrhage. **Type 4:** CABG-related bleeding. **Type 5a:** probable fatal bleeding. **Type 5b:** definite fatal bleeding.

BARC, bleeding academic research consortium; GRACE, global registry of acute coronary events; GUSTO, global use of strategies to open occluded arteries; Hgb, hemoglobin; PRBC, packed red blood cell; REPLACE-2, randomized evaluation in PCI linking angiomax to reduced clinical events; TIMI, thrombolysis in myocardial Infarction.

The prognostic implications of major bleeding complications are only further amplified in patients undergoing large bore access for MCS devices or structural heart interventions, often with delivery catheters that are 14 Fr or greater in diameter (23–25). In the case of the former, they are frequently inserted under emergency circumstances when patients are experiencing circulatory collapse (8). These findings were noted in recent observational data from the Cath-PCI and Chest Pain-MI registries which showed that among 1,680 propensity matched pair patients with acute myocardial infarction complicated by CS undergoing PCI, those supported with axial-flow percutaneous ventricular assist devices (pVAD) had double the risk of bleeding with more than 10-fold increased risk of death compared to patients supported with smaller platform intra-aortic balloon pumps (23). Frequently performed under more elective circumstances, patients undergoing structural heart interventions are similarly at risk for access site complications. While there has a notable decline from the nearly 15% incidence rates of periprocedural bleeding and vascular complications seen in the early days of TAVR due to smaller 14 Fr sheaths delivery systems, the frequency of these adverse events and variations in clinical expertise around recognition and management remain significant (26, 27). A contemporary analysis of 34,893 patients undergoing TAVR from the Society of Thoracic Surgeons/American College of Cardiology Transcatheter Valve Therapy (TVT) Registry from 2011 to 2016 observed a 7.6% incidence of in-hospital bleeding events (28). Significant inter-hospital variations were noted, with complication rates ranging from 0% to 46% among the 345 TVT sites (28). A bleeding complication was associated with significant increased risk for death and hospital readmissions at 1 year (28).

### Transradial access

Since its first description by Lucien Campeau in 1989, the adoption of transradial access (TRA) has steadily increased worldwide (29–31). It is associated with reductions in bleeding and vascular complications as well as major adverse cardiac events compared to transfemoral access (TFA) across the spectrum of coronary artery disease (32). It is also associated with improved quality-of-life outcomes, with reductions in bed rest time and bodily pains (33, 34). There are also potential economic benefits to TRA, with a significant reduction in healthcare costs related to not only fewer access site complications, but also shorter hospital lengths of stay, with nearly 20% increased likelihood for same-day discharge following elective PCI (35–37). A contemporary analysis of 279,987 PCI patients observed a nearly $3,700 adjusted savings in hospital costs in patients undergoing TRA for PCI who were discharged home the same day (36). With advances in radial sheaths and associated catheter technologies, TRA is also feasible in high-risk patients with complex coronary anatomy or hemodynamic compromise, where larger sheath sizes may be needed to facilitate intervention. In these circumstances, TRA is feasible without any appreciable increased risk for bleeding/vascular complications, contrast or radiation exposure (38). Given these considerations, TRA is recommended as the default access site for patients undergoing diagnostic coronary angiography and PCI (39–41).

### Bleeding and vascular access site complications with TRA

Initial observational studies with TRA demonstrated lower rates of major and minor bleeding compared to TFA, particularly in high-risk subgroups (42, 43). A retrospective cohort study of 2,820,874 PCI procedures from the National Cardiovascular Data Registry from 2007 to 2012 demonstrated that while TRA accounted for only 1 in 6 PCI's, it was associated with a significantly lower adjusted risk of bleeding (aOR 0.51; 95% CI: 0.49–0.54; *p *< 0.001) and vascular complications (aOR 0.39; 95% CI: 0.31–0.50; *p *< 0.001) compared to TFA, with the greatest benefit seen in women, patients ≥75 years of age and those with ACS (42). Similar finding were noted in the UK where TRA was adopted earlier, with marked reductions in major adverse cardiac events in patients with both stable ischemic heart disease and acute coronary syndrome (ACS) (35, 44–46).

The Radial vs. Femoral Access for Coronary Intervention (RIVAL) trial initially showed the greatest bleeding reductions among centers with the highest TRA utilization rates, suggesting that there may be volume-outcome relationship with utilizing the radial artery (44). However, subsequent studies including Radial vs. Femoral Randomized Investigation in ST-Elevation Acute Coronary Syndrome (RIFLE-STEACS) and Minimizing Adverse Haemorrhagic Events by TRansradial Access Site and Systematic Implementation of angioX (MATRIX), further confirmed these findings across the spectrum of TRA-expertise with reductions in composite endpoints encapsulating outcomes such as death, MI, stroke, recurrent MI and target lesion revascularization (35, 46). Subsequent investigations, including a meta-analysis of 24 studies and a *post-hoc* examination of a high volume North American quaternary care center, have subsequently confirmed these findings with reductions in major adverse cardiac events compared to TFA across the severity spectrum of CAD, including cardiogenic shock (32, 47). Interestingly, the most recently published study in this field, The Safety and Efficacy of Femoral Access Versus Radial for Primary Percutaneous Coronary Intervention in ST-Elevation Myocardial Infarction (SAFARI-STEM) trial, was terminated early due to futility as it failed to demonstrate the superiority of TRA in STEMI with regards to 30-day all-cause mortality, non-surgical TIMI major bleeding, or death/re-infarction/stroke compared to TFA in patients with STEMI (48). While this study has been scrutinized for its limited statistical power and for enrolling patients with lower acuity of illness and bleeding risk than previous trials, its findings do highlight the need for the development of institutional competency pathways in vascular access so that operators may remain facile with both access techniques in contemporary clinical practice. A summary of the pertinent RCT's evaluating TRA is highlighted in Table 2.

**Table 2:** Clinical trials comparing radial versus femoral arterial access in acute coronary syndrome.

Study name	RIVAL (44)	RIFLE-STEACS (35)	RIVAL-STEMI (45)	MATRIX (46)	SAFARI-PCI (48)
Study Design	Multicenter, 32 countries. 1:1 randomization; Open label	Multicenter, European, 1:1 randomization; Open label. 4 high volume radial centers	Multicenter, 32 countries, 1:1 randomized, open label.	Multicenter, European centers; 1:1 randomization; 0pen Label	Multicenter, 5 PCI centers in Canada. Randomized, open label.
Population	7,021 patients with ACS	1,001 patients with STEMI	1,958 patients with STEMI	8,404 patients with ACS	2,292 STEMI patients
Study arms	Radial vs. Femoral	Radial vs. Femoral	Radial vs. Femoral	Radial vs. Femoral	Radial vs. Femoral
Outcomes	- Composite of death, MI, stroke, or bleeding: 3.7% vs. 4%. *P* = 0.50 - Vascular complications including pseudoaneurysm, hematoma, fistula, ischemic limb: 1.4% vs. 3.7%. *P* < 0.0001	- Composite of CV death, recurrent MI, CVA, TLR or bleeding: 13.6% Vs 21%; *p *= 0.003	- Composite of death, MI, CVA or bleeding: 3.1% vs. 5.2%. *P* = 0.026 - Vascular complications: 1.3% vs. 3.4%. *P* = 0.002	- Co-primary composite endpoints of 1. Death, MI or stroke: 8.8% TRA vs. 10.3% TFA; *p *= 0.0307. 1. Death, MI, stroke, or BARC non-CABG major bleeding: 9.8% TRA vs. 11.7% TFA; *p *= 0.0092.	- 30-day all-cause mortality: 1.5% vs. 1.3%. *P* = 0.69

ACS, acute coronary syndrome; BARC, bleeding academic research consortium; CABG, coronary artery bypass graft surgery; CVA, cerebrovascular access; MATRIX, minimizing adverse haemorrhagic events by transradial access site and systemic implementation of AngioX; MI, myocardial infarction; RIFLE-STEACS, Radial versus femoral randomized investigation in ST-segment elevation acute coronary syndrome; RIVAL, radial versus femoral access for coronary angiography and intervention in patients with acute coronary ssyndromes; SAFARI STEMI, safety and efficacy of femoral access vs radial access in ST-elevation myocardial infarction; STEMI, ST-elevation myocardial infarction; TFA, transfemoral access; TRA, transradial arterial access; TLR, target lesion revascularization.

Access site complications associated with TRA occur infrequently, between 0.2%–1.0% of cases (32). While rare, they can be associated with significant morbidity. Thus it is important to recognize and manage these sequelae in timely fashion. Vascular complications which may be seen following TRA include radial artery spasm, perforation, occlusion due to intimal medical hyperplasia, arteriovenous fistulae and forearm hematomas with compartment syndrome (49). Like most cardiac procedures, there is a learning curve with accessing the radial artery safely and effectively. It is recommended that operators learning TRA perform at least 50 cases initially followed by a minimum of 80 procedures annually to achieve and maintain proficiency, respectively (50). It has also been demonstrated that operators facile with TRA are able to perform both diagnostic cardiac catheterizations and coronary interventions with similar efficacy and safety vs. femoral access, as demonstrated by comparable radiation dosages and total fluoroscopy/procedural times in high-radial volume centers (48, 51).

### Best practices for transradial arterial access and hemostasis

A “radial-first” approach is strongly encouraged for all-comers undergoing coronary angiography or PCI (39). The radial artery however is small, typically measuring 2.2 mm–2.6 mm in diameter, and inability to successfully cannulate the vessel is a major contributor to nearly 60% of cases in which there is TRA failure (52). The radial approach can also be challenging in patients with systemic vasculitides, such as Raynaud's disease, as well as in patients with chronic kidney disease due to calcified vessels, brachiocephalic tortuosity, and in those with prior ipsilateral mastectomies with lymph node dissection (39). In the case of the latter, caution against TRA has been borne out of concern for potential risk of access site infection and lymphedema, although this has not been seen in observational studies (53). During TRA, the radial artery is subject to trauma resulting from intimal tears, medial dissection, long-term intimal medial thickness, and impaired flow-mediated vasodilation (54–56). These acute and chronic changes in turn may compromise its utility to serve as a suitable conduit for future coronary artery bypass grafting (CABG) and non-cardiac surgeries, such as arteriovenous fistula formation (56). While not an absolute contraindication to TRA, it is advised to avoid using the ipsilateral radial artery for CABG for at least 3 months given observational data demonstrating reduced radial artery bypass graft patency (3). Historically, confirmation of dual arterial supply to the palmar arch had been required prior to TRA, by examining pulse oximetric waveform patterns following manual occlusion of the ipsilateral radial artery. Commonly referred to as the Barbeau or Allen exams, these pre-procedural tests were once widely performed to predict the risk of hand ischemia. However, given a recent study failing to demonstrate significant differences in thumb capillary lactate, hand grip strength or discomfort between patients with normal and abnormal Barbeau exams, routine pre-procedural assessment of collateral hand circulation is no longer recommended and should not preclude TRA (3, 57). While most interventionalists prefer right TRA primarily because of ergonomic purposes, consideration may be given to left TRA in certain cases. These include patients with brachiocephalic tortuosity and those who have undergone CABG with left internal mammary arteries, although right TRA may still be feasible in these circumstances (58–61). The use of two-dimensional ultrasound may facilitate more timely radial arterial puncture by allowing for direct visual inspection of vessel size, potential anatomic anomalies, needle tip puncture, wire passage and sheath insertion. Several studies have published the benefits associated with ultrasound-guided TRA, including a meta-analysis of 12 studies (*n* = 1,992) and the Radial Artery Access with Ultrasound Trial (RAUST), a multi-center RCT of 698 patients undergoing TRA who were randomized to manual palpation or ultrasound guidance (62, 63). Both studies showed enhanced efficiency and success with TRA when US was utilized, with the former also reporting reductions in site hematomas, findings which result in reductions radial artery occlusion (RAO) up to 30 days post-procedure (62–64). TRA may be obtained either by way of single- or double-wall puncture, with the latter technique associated with higher rates of successful puncture on the first attempt (65). In cases in which TRA is not successful, the ipsilateral brachial or ulnar arteries may be considered as alternative conduits. Limited studies have demonstrated comparable safety and efficacy between the radial and ulnar access sites, although the ulnar approach was associated with increased access site cross-over (66, 67). While not observed in clinical studies, caution is advised with ulnar access in patients with occluded ipsilateral radial arteries given the risk for hand ischemia with ulnar nerve injury (68).

During TRA, concerted efforts should also be made to undertake measures to prevent radial artery occlusion (RAO), a multifactorial phenomenon stemming from vascular injury and inflammatory changes of arterial wall during sheath insertion. Procedural factors associated with RAO include insufficient anticoagulation, sheath-to-arterial diameter ratio >1, multiple puncture attempts, vasospasm and prolonged occlusive hemostasis (64, 69–74). Pharmacologic measures to achieve this goal include adequate administration of moderate sedation and local analgesia, anti-spasmolytic therapy with intra-arterial nitroglycerine (100–200 *μ*g) and verapamil (5 mg), as well as systemic anticoagulation with intra-arterial or intravenous heparin (at least 5,000 U or 50 U/kg bolus) following sheath insertion (69, 70, 75). Iterative advances in TRA technology with reductions in sheath size and hydrophilic coating have also advanced the field, allowing operators to perform complex PCI via TRA with relative impunity. An example of this advancement is the 6 Fr Glidesheath Slender (Terumo, Somerset, NJ), a novel thin-walled sheath design with 5 Fr (2.46 mm) outer diameter while maintaining 6 Fr inner architecture. This technology was compared in a randomized, multi-center non-inferiority trial with standard 5 Fr technology, and no differences were noted with regards to RAO, vasospasm, access-site vascular complications or local bleeding (71). Cordis (Rainsheath) and Merit Medical (Prelude Ideal) similarly have thin-walled sheath platforms which can facilitate complex PCI using 7 Fr guide catheters. An alternative conduit for TRA coronary angiography and PCI are sheathless systems, which are usually 1–2 Fr smaller than standard sheaths and obviate the need for additional mechanical stretch of the radial artery (76). Two commonly used sheathless platforms are the Eucath (Asahi, Irvine, CA) and the Railway (Cordis, Hialeah, Fl) systems. They are introduced using a dilator and without the need for a sheath, and while 5 Fr, 6 Fr, or 7 Fr catheters can be used with these technologies, catheter backup and manipulation may not be as consistent as with traditional sheathed systems. Sheathless guide catheters have been evaluated in non-randomized cohort studies with successful outcomes in patients with calcified and bifurcation lesions (77, 78). While there have not been any randomized clinical trials studying thin-wall sheaths and sheathless platforms for PCI in head-to-head fashion, a recent propensity-matched analysis of 728 patients with acute coronary syndrome, those undergoing transradial PCI using a 7 Fr Glidesheath Slender had lower rates of RAO (OR = 0.32, 95% CI: 0.11–0.93; *p *= 0.036) (79) Further research is needed to better understand the optimal transradial platform for performing safe and effective higher risk PCI in patients with complex CAD. A summary of currently available transradial sheaths and sheathless systems is described in Table 3 (80).

**Table 3:** Current transradial sheaths and sheathless catheter platforms.

Transradial platform	Sheath size	Outer diameter
Standard sheath	6 Fr	2.65 mm
** **	7 Fr	2.95 mm
** **	8 Fr	3.25 mm
GlideSheath slender	6 Fr	2.46 mm
** **	7 Fr	2.80 mm
Eaucath sheathless	6.5 Fr	2.16 mm
** **	7.5 Fr	2.49 mm

Upon completion of the cardiac catheterization, steps should be undertaken to ensure safe and effective hemostasis. A number of radial occlusion devices are currently available for commercial use. To date, a strategy of patent hemostasis has been shown to be most effective in reducing the rates of RAO, with the Prevention of Radial Artery Occlusion—Patent Hemostasis Evaluation (PROPHET) study demonstrating reductions in early (<24 h) and late (30 days) incidence of RAO of 59% and 75%, respectively, compared to a strategy of conventional pressure application (73). Shorter durations of hemostatic compression have been associated with reductions in radial artery compromise, with many centers employing protocols which advocate for patent hemostasis times of 2 and 4 h following diagnostic angiography and PCI, respectively (69). Prophylactic ipsilateral ulnar artery compression following completion of transradial cardiac catheterization has also been shown to be efficacious in not only facilitating safe and patent hemostasis of the RA, but also in reducing the risk of RAO. The PROPHET-II (PROPhylactic Hyperperfusion Evaluation Trial), a multicenter randomized clinical trial of 3,000 patients undergoing TRA for cardiac catheterization demonstrated that a strategy of occlusive compression of the ipsilateral ulnar artery at the Guyon's canal at the time of radial band application followed by removal of the radial sheath and patent hemostasis was associated with nearly 3-fold reduction in RAO up to 30-days post-procedure compared to patent hemostasis alone (81). It is recommended that TRA centers develop post-procedural care pathways and quality assurance mechanisms to ensure that multi-modality assessments are employed to ensure timely hemostasis and early ambulation following transradial coronary angiography or PCI (39).

### Distal transradial access

More recently, distal transradial access (dTRA) in the “anatomic snuffbox” a triangular depression on the dorsum of the hand that is bordered by the extensor pollicis brevis and abductor pollicis longus tendons laterally and by the extensor pollicis longus tendon medially, has been proposed as an alternative cannulation site to traditional TRA in the wrist (82). In addition to ergonomic benefits and shorter hemostasis times due to the superficial location of the distal RA with a boney basement in the snuffbox, dTRA may also potentially impart greater reductions in RAO compared to traditional TRA (83, 84). The putative mechanism for this hypothesis is the more distal location of the puncture site, which in turn may facilitate greater preservation of flow in the radial artery proximally, due to the rich cascade of adjacent collateral networks from the deep palmar arch. The safety and feasibility of dTRA for coronary angiography and PCI has been demonstrated, with clinical trial data suggesting greater reductions in RAO, as determined by doppler ultrasound, compared to traditional TRA (84, 85). Little is known, however, regarding the pathobiology of radial artery injury and remodeling following dTRA. The PRESERVE Radial study (A PRospEctive Randomized Clinical Study Comparing Radial ArtERy Intimal Hyperplasia Following Distal Vs. ForEarm TransRADIAL Arterial Access for Coronary Angiography) (NCT04801901) will be the first clinical trial to compare intimal medial hyperplasia and other markers of vessel healing following dTRA and traditional TRA using ultrahigh resolution ultrasound. Given the more superficial location and potential tortuosity of the radial artery in the snuffbox, however, patients undergoing dTRA may experience greater number of puncture attempts, time-to-sheath insertion and access-site crossover (84, 86).

### Transfemoral access

**Figure 2:**
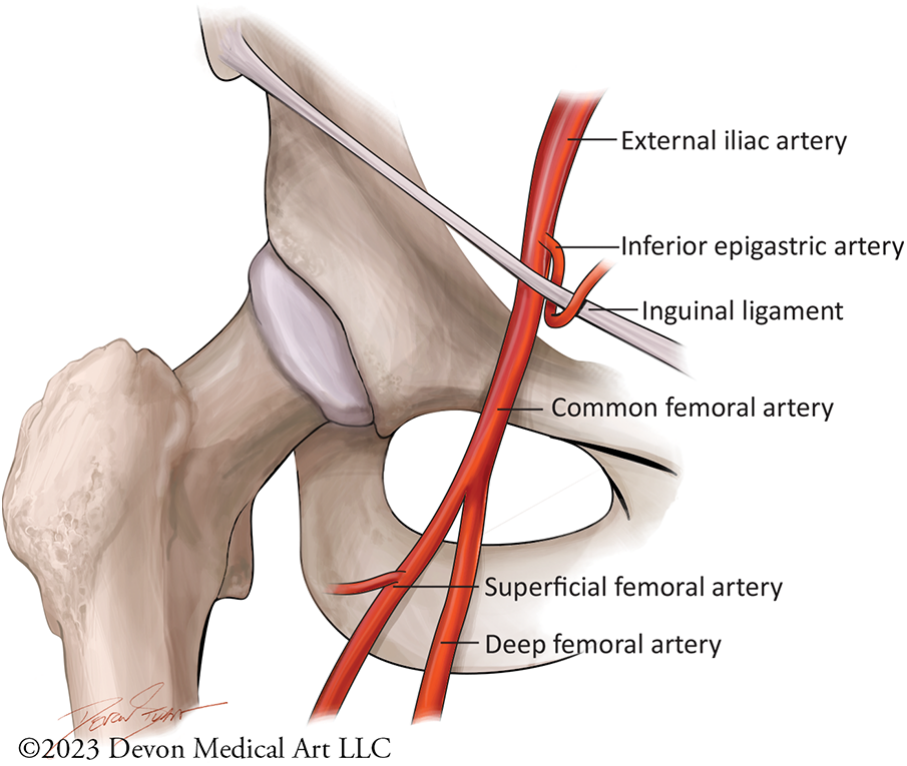
Femoral artery anatomy. This cartoon figure depiction demonstrates the branches of the femoral artery. Safe and effective transfemoral access should ideally rely on fluorscopic and ultrasound-guided landmarks, with micropuncture access of the common femoral artery at the lower edge of the femoral ahead below the inferior epigastric artery.

Despite a significant shift towards TRA for coronary angiography and PCI, the femoral artery remains a necessary conduit for numerous procedures in contemporary clinical practice, such as the insertion of percutaneous MCS devices and TAVR. It is still also required in non-large bore coronary angiogram/PCI cases (<8 Fr) where TRA or ulnar access are not feasible. Contemporary TFA should ideally employ four multimodality techniques: 1. Fluoroscopy; 2. Doppler ultrasound; 3. Micropuncture needles; and 4. Iliofemoral angiography (87). Familiarity with femoral artery anatomy is key to safe and efficient vascular access and closure. The Common Femoral Artery (CFA) is a continuation of the External Iliac Artery and lies below the inferior epigastric artery, the anatomic location of the inguinal ligament, where the vessel dives posteriorly into the retroperitoneal space. Distally, the CFA bifurcates into the Superficial Femoral Artery (SFA) and Profunda Femoris Artery (PFA). The angiographic anatomy of the femoral arteries are shown in Figure 2. The following steps are recommended for optimal TFA (87):

1. The inferomedial edge of the femoral head should be identified under fluoroscopy and its position should be noted either by way of a hemostat or marker.
2. Ultrasound should then be used to scan for the CFA and its tributaries.
3. Local anesthetic should then be applied using ultrasound guidance to the subcutaneous tissue above and adjacent to the CFA.
4. Using ultrasound guidance, a 21-gauge micropuncture needle should then be used to access the CFA approximately 1 cm–3 cm below the probe with direct visualization of the puncture. This is recommended to avoid a steep angle on the needle with consequent risk for high femoral access, and thus a retroperitoneal bleed. A micropuncture needle may be preferrable to standard 18 gauge platform as it can reduce not only the size of the initial puncture, but also the amount of bleeding associated with vascular injury should there be multiple attempts at cannulating the artery (8). Despite these potential advantages, randomized data supporting this strategy is lacking, and it is advised that institutions develop best practices around vessel puncture, regardless of which needle platform is used (88).
5. Once TFA is obtained, a 0.018″ micro access guidewire should then be advanced under fluoroscopy to ensure appropriate passage, avoiding the circumflex iliac artery, as inadvertent perforation of this vessel with a guidewire may lead to an abdominal hematoma, requiring blood transfusion and endovascular intervention (89).
6. A 4 Fr microcatheter dilator should then be advanced over the guidewire. An iliofemoral angiogram may be performed through the microcatheter dilator, typically at 30 degree ipsilateral angulation, to confirm the location of the arteriotomy. If the access site is not felt to be appropriate, the dilator may then be removed followed by 10 min of manual pressure prior to re-access. Low access at the level of the superficial femoral artery is associated with increased risk for arteriovenous fistula formation, pseudoaneurysms and thrombosis. If the arteriotomy site is deemed to be satisfactory, the tract can then be dilated to place the requisite sheath size to perform the respective cardiac procedure.

### Ultrasound guided femoral artery access

**Figure 3:**
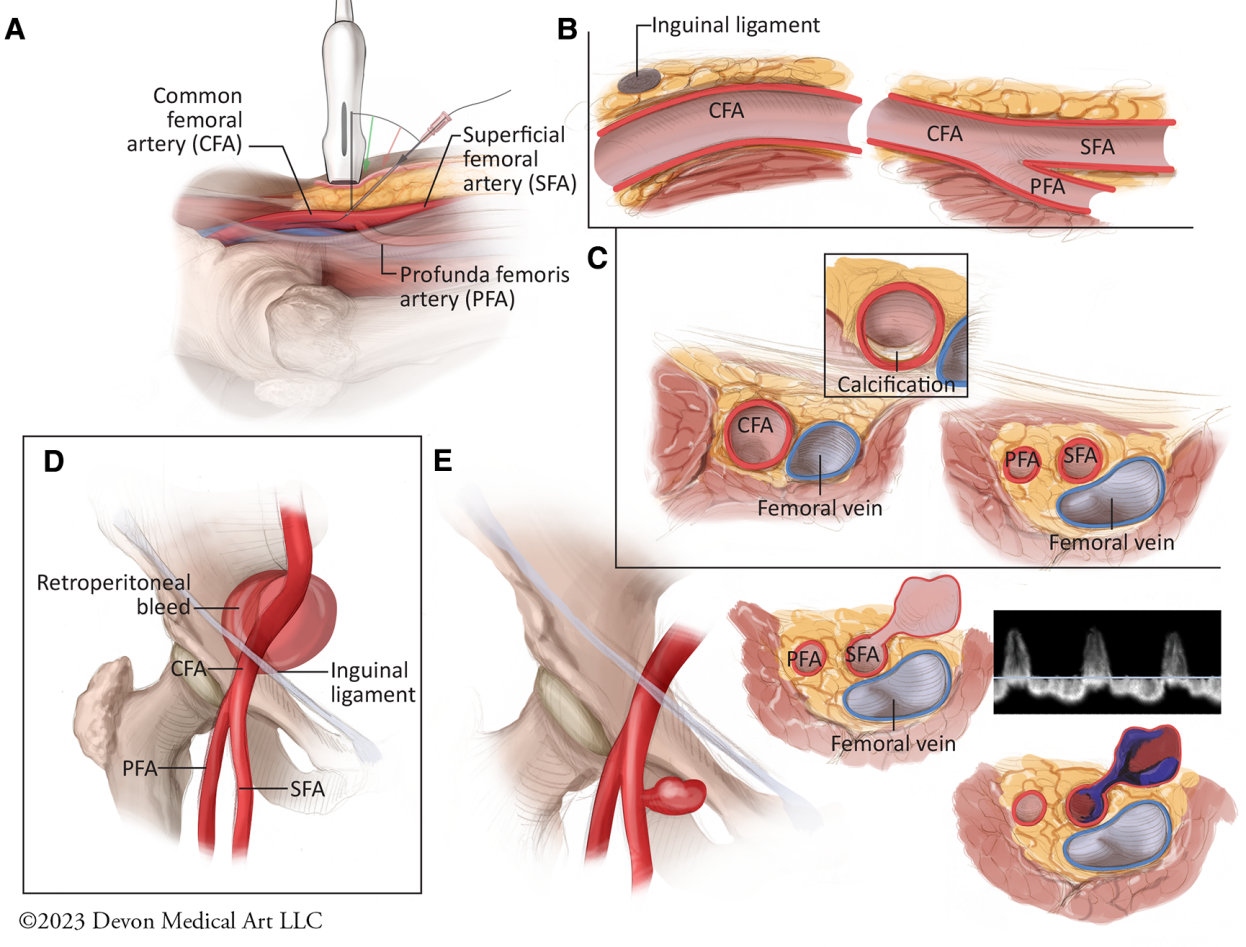
Best practices for femoral arterial access and potential vascular complications. (**A**) The optimal technique for ultrasound-guided femoral arterial access requires the use of a 5- to 10-MHz linear ultrasound probe which is held such that the vertical marker is facing upward from the femoral artery to facilitate a longitudinal view of the common femoral artery and its branches. The access needle should then be used at a 45 degree angle to access the common femoral artery. (**B**) Longitudinal view of the femoral vasculature demonstrates the posterior dive that the common femoral artery takes below the inguinal ligament as the probe is advanced cranially. (**C**) Rotating the ultrasound proble 90 degrees allows for cross sectional view of the femoral bifurcation, including anterior or posterior vessel wall calcification. (**D**) Vessel puncture cranial to the inferior epigastric artery is associated with increased risk for retroperitoneal bleeds. (**E**) Vessel puncture below the common femoral artery bifurcation and involving the superfical femoral artery is associated with increased for pseudoaneurysm formation. Pseudoaneurysms can present clinically as pulsatile hematomas, painful ecchmyoses or as frank bleeding. They typically consist of 1 or 2 layers of the vessel wall, and are therefore associated with increased risk for rupture, embolization, and limb ischemia. They typically demonstrate “to-and-fro” patterns of flow with pulsed Doppler assessment.

Routine utilization of ultrasound should be the standard of care for all TFA cases for optimal access and preventing the risk for vascular complications (Figure 3) (87). The merits of ultrasound guidance for femoral access have been studied extensively, including a meta-analysis of 1,422 patients demonstrating nearly 50% reduction in all complications, including bleeding and accidental puncture, as well as 42% improvement in likelihood of successful arterial access on the first attempt (90–93). The Femoral Arterial Access with Ultrasound Trial (FAUST) demonstrated that the use of ultrasound was associated with 80% increased likelihood of first-pass success, 85% reduced risk of accidental venipuncture and nearly 60% reduction vascular complications compared to fluoroscopic guidance (90). The greatest benefit with successful vessel cannulation was noted with common femoral arterial bifurcations above the femoral head, an inherently high risk cohort of patients (90). Interestingly, the routine Ultrasound Guidance for Vascular Access for Cardiac Procedures (UNIVERSAL) Trial, a multi-center prospective study 621 patients undergoing TFA for coronary angiography or PCI, failed to show a decrement in Bleeding Academic Research Consortium 2, 3, or 5 major bleeding or vascular complications with ultrasound-guided access compared with manual palpation, although the former was associated with reduced number of access attempts and accidental venipunctures (94). While the study has been critiqued for not mandating the use of micro puncture catheters and for including BARC 2 minor bleeds which may be unrelated to the procedure, the findings do highlight the need for further research in the form of large multicenter registries and clinical trials evaluating the potential benefits of skilled ultrasound utilization to reduce the risk of adverse periprocedural sequelae during TFA (95).

### Vascular closure devices

Historically, manual compression has been the standard of care for achieving hemostasis following femoral arterial sheath removal, usually once activated clotting time (ACT) has been less than160–180 s (96). Given the labor intensive nature of digital compression by catheterization laboratory staff members, a number of mechanical compression devices, such as the Femostop (Radi Medical System) and CompressAR C-clamp (Advanced Vascular Dynamics) were subsequently developed, with some studies suggesting that they may be associated with reduced vascular complications compared with manual hold (97–99). Despite the benefits of allowing staff members to be relieved from the demands of bedside manual compression, these devices do need careful supervision to ensure that they do not migrate and that they are not applied at pressures which could compromise limb flow and result in arterial and/or venous thrombosis.

In response to the logistical challenges associated with manual pressure and passive closure platforms, a variety of vascular devices (VCD) have been developed since the mid 1990s which can be deployed at the conclusion of cardiac catheterization, allowing for potentially faster hemostasis, shorter duration of bed rest and reductions in patient discomfort (96). They are used in up to 75% of TFA cases in contemporary clinical practice (100). VCD's have historically been classified into 4 categories: plugs, sutures, glues or topical patches. Despite their potential to improve patient satisfaction and clinical throughput, the evidence supporting their use has been limited to observational studies and modest sized clinical trials (100). These studies have demonstrated the safety and feasibility of VCD's, but they have not provided definitive evidence around efficacy or reductions in bleeding complications compared to manual compression (100–105). As such, caution is advised with the routine deployment of these devices following TFA. Instead, societal guidelines advocate for a patent-centered approach that takes into consideration clinical factors such as body habitus, arteriotomy location and vessel anatomy (size, calcification tortuosity) (101). It is advised that all patients undergoing cardiac procedures via TFA undergo femoral angiography prior to closure to determine whether the arteriotomy site and vessel anatomy are suitable for the deployment of these devices. Potential complications associated with these devices include acute limb ischemia, bleeding, infection, distal embolization, pseudoaneurysm and arteriovenous fistula formation (106, 107). It is recommended that interventionalists have familiarity with all device and that they able to address any potential device-related complications with facile expertise (108). Table 4 provides a summary of the some of the currently Food and Drug Administration approved VCD's employed in clinical practice in the United States.

**Table 4:** Most currently employed vascular closure devices in the United States.

Device	Proglide	Angio-Seal	StarClose	Mynx	ExoSeal	FISH	MANTA
Manufacturer	Abbott	Terumo	Abbott	Cordis	Cordis	Morris Innovative	Teleflex
Mechanism	Suture	Collagen and suture	Clip (nitinol)	Hydrogel Plug (PEG)	Plug (Polyglycolic acid)	Bioabsorbable extracellular matrix ribbon	Collagen-based for large bore access
Sheath size	6 Fr	6 Fr–8 Fr	5 Fr–6 Fr	5 Fr–7 Fr	5 Fr–7 Fr	5 Fr–8 Fr	14 Fr and 18 Fr
Re-access within 90 days	Yes	In close proximity (1 cm higher)	Unknown	Yes	Unknown	2 cm above the previous access	Unknown
Placement	Extraluminal	Intraluminal	Extraluminal	Extraluminal	Extraluminal	Intraluminal	Intraluminal

The Angio-Seal system (Terumo, Somerset, NJ) is one of the most commonly used platforms in the United States, consisting of a co-polymer which is deployed intravascularly and attached by an absorbable Dexon traction suture to an extravascular collagen plug (96). It was first studied against manual compression in a multi-center RCT of 435 patients undergoing cardiac catheterization and was noted to have 85% reduction in time to hemostasis and lower rates of bleeding and hematomas (109). These results were further observed in a subsequent clinical trial of 612 patients undergoing PCI who were randomized to Angio-Seal or manual compression (110). These patients had select high risk features for vascular complications (age >70, previous puncture at the same site, hypertension, treatment with ticlopidine at least 2 days prior, use of abxicimab, 8 Fr access, prolonged heparin treatment after the angioplasty, and use of fibrinolytics) (110). Angio-Seal was associated with significant reductions in time to hemostasis, total bedrest time, and bleeding (110). Available in 6 and 8 Fr platforms, Angio-Seal is rapidly deployable with hemostasis success rates of up to 97% (107).

**Figure 4:**
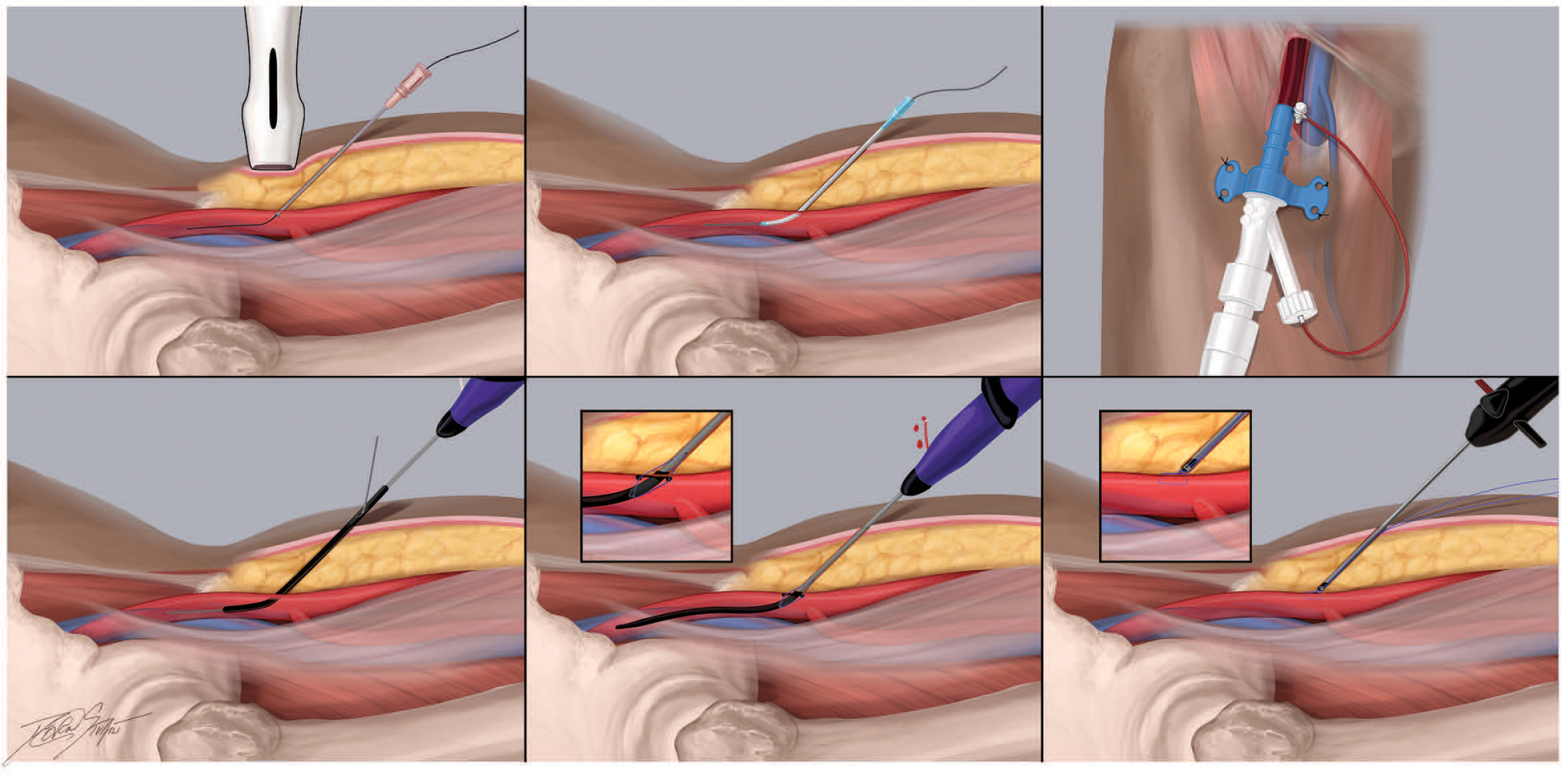
Recommended practices for the perclose proglide vascular closure device. The Perclose Proglide platform (Abbott, Chicago, IL) is a suture-mediated vascular closure device commonly used in contemporary practice to hemostase standard and large bore femoral arterial access sites. It consists of two needles in the proximal compartment and a catheter that house the suture. It is 0.035″ J wire compatible. This cartoon depiction demonstrates the steps of large bore access and closure using this platform. (**A**) Using ultrasound guidance, the common femoral artery is accessed with a 21 gauge micrpuncture needle 1-3 cm below the probe. (**B**) Once femoral access is obtained, a.018″ micro access guidewire should then be advanced under fluoroscopy to ensure appropriate passage into the ipsilateral common iliac artery and abdominal aorta. (**C**) Following confirmation of the femoral access site, the arteriotomy may be “pre-closed” using 1 or 2 Proglide sutures, typically in the 10- and 12-o’clock positions. Here, the percutaneous ventricular assist device is secured in place following dilation of the arteriotomy (**D**) During deployment of the Proglide system, the catheter is advanced over an 0.035″ J wire through the arteriotomy. (**E-F**) Once the return of pulsatile blood is noted from the side arm, the device lever is pulled and two simultaneous needles are deployed through the anterior wall of the femoral artery utilizing the non-biodegradable polypropylene microfilament with preformed knot that is tightened to form the suture loop. The suture is then retracted to the anterior wall of the arteriotomy site and deployed to achieve hemostasis.

The ProGlide Perclose device (Abbott Vascular, Abbott Park, IL) is a suture-mediated device that is advanced over an 0.035″ J wire through the arteriotomy after the femoral sheath is removed. Once the return of pulsatile blood is noted from the side arm, the device lever is pulled and two simultaneous needles are deployed through the anterior wall of the femoral artery utilizing the non-biodegradable polypropylene microfilament with preformed knot that is tightened to form the suture loop. The suture is then retracted to the anterior wall of the arteriotomy site and deployed to achieve hemostasis (96) (Figure 4). The Perclose platform is unique among VCDs as it maintains access to the vessel by way of the 0.035 inch guidewire after the needles and suture have been deployed. Therefore if the needles do not capture the sutures and adequate hemostasis is not achieved, the guidewire may be re-introduced into the arteriotomy and ultimately removed following confirmation of satisfactory hemostasis. Similar to the Angio-Seal device, Perclose has been associated with reduced time to hemostasis and ambulation with no significant differences in bleeding complications compared to manual hemostasis (111, 112). In experienced hands, it can also be associated with significant costs savings as it may obviate the need for further manpower and work personnel typically associated with manual compression (113).

The MANTA™ vascular closure received pre-market investigational device exemption approval by the Food and Drug Administration in 2019 following multi-center observational data demonstrating safety and feasibility in 263 patients undergoing TAVR and percutaneous endovascular thoracic and abdominal aortic aneurysm repair (114). With an effective sheath outer diameter of 22 Fr or 7.3 mm, rapid closure was able to be achieved, with median time to hemostasis of 24 s and only 4.2% rate of Valve Academic Research Consortium-2 major vascular complications (114). A collagen-based platform, it is currently available in 14 Fr and 18 Fr configurations, and it is 0.035″ guidewire compatible, consisting of a poly-lactic-co-glycolic snap attached to a bovine collagen plug which is tampered down, sandwiching the arteriotomy while a toggle is deployed intravascularly. The toggle and collagen plug usually are fully absorbed within 6 months and the vessel may be re-accessed 2.5 cm cranial or caudal to the MANTA device (96). The safety and efficacy of MANTA was recently compared with Proglide in the Randomized Comparison of Catheter-based Strategies for Interventional Access Site Closure during Transfemoral Transcatheter Aortic Valve Implantation (CHOICE-CLOSURE) study, a multi-center RCT of 516 patients undergoing TAVR (115). The authors reported significantly lower times to hemostasis with MANTA [80 (32–180) vs. 240 (174–316) seconds; *p *< 0.001) but at the cost of increased rates of access-site vascular complications [relative risk, 1.61 (95% CI: 1.07–2.44), *p *= 0.029], in particular pseudoaneurysms and hematomas (115). No differences were noted, however, with respect to access site bleeding or device failure. Historically, vascular closure following TAVR and other large bore access procedures has been performed by tightening the knots of 1 or 2 Perclose devices which were partially deployed at the time of initial access (“pre-closure”) (116).Further research is needed in the form of large multi-center registries and nested clinical trials using standardized best-practices to identify closure strategies in patients undergoing large bore access that are the most safe, effective and cost-sensitive.

### Large bore vascular access and closure

The employment of safe and effective femoral arterial access is of paramount importance in patients requiring large bore sheaths (>8 Fr) for structural heart interventions, endovascular aneurysm repairs and percutaneous MCS placement. Despite advances in device-based therapies to treat these conditions using minimally invasive techniques, there remain significant variations in the technical expertise of large bore access within and among institutions (6, 8). A contemporary analysis of 17,672 patients undergoing large bore access for TAVR, EVAR and percutaneous MCS observed an 18% rate of bleeding complications, which in turn was associated with nearly 3-fold increased risk for in-hospital mortality, 2-fold increase in hospital-length-of-stay and marked increases in total healthcare costs (25). In cases requiring MCS implantation under emergency circumstances, the rates of bleeding and vascular complications can be as high as 40%, with greater than 4-fold increased risk for acute ischemia if advanced platforms such as percutaneous ventricular assist devices or VA-ECMO are utilized (117, 118). A summary of currently available MCS and structural heart platforms with their respective sheath sizes is described in Table 5. Given these considerations, societal recommendations and position statements have been developed around best practices for proper large bore femoral arterial access and closure (8, 119):

1. Similar to standard TFA, multi-modality imaging techniques should be employed to visualize the common femoral artery, including bifurcation, calcification and dimensions, with a minimal diameter of 6 mm typically needed for sheath sizes of up to 18 Fr. In elective circumstances such as TAVR, pre-procedural computed tomography may also be performed to assess iliofemoral anatomy, in addition to other anatomic considerations, such as annular dimensions, valvular calcification and coronary artery height (120).
2. Using fluoroscopic landmarks and doppler ultrasound, a 21 gauge micro-puncture needle should be used to access the CFA, typically 2 cm–3 cm below the mid portion of the inguinal ligament and 1 cm lateral to the lower third of the femoral head (8, 90).
3. Similar to standard TFA, all patients should undergo femoral angiography following micro-puncture access, using standard or digital subtraction angiography (87). Once the femoral access site has been deemed to be suitable, the arteriotomy tract should then dilated for placement of a 6 Fr sheath.
4. Given the clinical ramifications of bleeding and vascular complications associated with large bore access, it is advised that pre-procedural planning occur and contingency measures be developed around closure strategies. While it is generally recognized that VCDs may be preferrable to manual pressure following large bore access, there is no single best practice strategy around a particular device and when to employ it. A “pre-closure” strategy of using 1 or 2 Proglide sutures which are partially deployed, typically in the 10- and 2-o'clock configurations, is commonly employed in contemporary practice, and has been associated with low rates of bleeding and vascular complications (116).
5. Given the enhanced complexities and time intensive nature of large bore procedures, it is advised that operators make concerted effort to mitigate radiation exposure to the patient and staff members at all times. This includes at the time of access when inguinal fluoroscopy is performed to confirm device placement and vessel patency. Strategies include ensuring that the patient is close to the image receptor and away from the x-ray source so as to reduce radiation scatter, maintaining at least 1 cm distance between the operator and the patient, avoiding the use of steep angles with the x-ray beam and placing the C-arm in 0° to 20° angulations, minimizing the number of cineograms and shortening each cine acquisition, utilizing lower frame rates (i.e. 7.5 frames/s) and the application of protective scatter-radiation absorbing shields (121, 122).
6. Once the arteriotomy has been duly upsized and the respective large-bore sheath has been secured, a run-off angiogram should be performed to ensure adequate distal limb perfusion (8). If limb flow is impaired, a distal perfusion catheter consisting of a 5- or 6-Fr braided sheath may be placed using ultrasound-guided antegrade access of the ipsilateral superficial or profunda femoral artery. Distal perfusion can be maintained by utilizing one of three donor strategies: (1) “External” bypass from the ipsilateral common femoral artery by connecting the side-arm of the large-bore sheath to the distal perfusion cannula; (2) “External” bypass from the contralateral common femoral artery by connecting the side-arm of a 5 or 6-Fr retrograde sheath placed in the contralateral vessel to the distal perfusion cannula; and (3) “Internal” contralateral bypass from a 7 Fr retrograde sheath in he the contralateral common femoral artery to the ipsilateral profunda femoris artery through an up-and-over internal 4 Fr sheath which is inserted through the contralateral conduit (8, 123). The radial artery may also be utilized as a donor for distal perfusion, although caution is advised if it prolonged indwelling of the radial sheath is anticipated, given the potential risk for radial artery thrombosis and upper extremity digital ischemia (8). It should be noted that the feasibility and safety for distal perfusion cannula placement is primarily from observational data, and further research in the form of randomized clinical trials is needed to inform the application of this practice on a larger scale (8, 119, 123).
7. While the radial artery has been demonstrated to reduce bleeding and major adverse cardiac events in patients undergoing complex PCI with pVAD support, there is an alternative technique currently employed at many centers in the United which may reduce the number of access sites. Commonly referred to the SHiP (Single access for High-risk PCI) technique, the procedure is facilitated by a micro-puncture needle which is used to pierce the diaphragm of the pVAD peel-away sheath, allowing for placement of a 7 Fr sheath through the hemostatic valve and adjacent to the 9 Fr pVAD catheter shaft, through which complex PCI can be performed (124).
8. Upon completion of the large bore cardiac procedure, it is recommended that vascular closure be performed in the cardiac catheterization laboratory or hybrid operating room with cardiac and vascular surgical back-up capabilities (8). Closure strategies currently employed in clinical practice include: 1) Deployment of the Perclose Proglides sutures (1 or 2) which were used to “pre-close” the arteriotomy at the onset of the case; (2) “Post-Closure” using a double-wire approach to facilitate the deployment of two Perclose Proglide; (3) “Hybrid” approach with the combined use of one Perclose Proglide suture and either one Angioseal or Mynx VCD; (4) “Dry closure” with balloon hemostasis of the ipsilateral external iliac artery via radial or contralateral femoral arterial access; or 5) Deployment of the MANTA device (114, 125–127).
9. Once hemostasis is achieved, final run-off femoral angiography (via the radial artery or contralateral femoral artery) is advised to ensure distal perfusion and recognize any potential vascular complications (8). In the event of vessel injury stemming from perforation, hemostasis can be obtained percutaneously by way of endovascular deployment of covered stents. While both self- and balloon-expandable platforms can be used, the former may be preferred given its more adaptive frame in response to daily physiologic stressors imposed on the iliofemoral vessels from flexion and extension (128).
10. All vascular complications stemming from standard or large bore TFA access should be reviewed at institutional quality assurances forums in standardized fashion by physician and administrative leadership. Direct feedback can then provided to the interventional team around potential opportunities for improvement (129).

**Table 5:** Current mechanical circulatory support platforms and structural heart devices.

Mechanical circulatory support	Cannula size
Intra-aortic balloon pump	7 Fr–8 Fr Arterial
Impella LV support (2.5, CP, 5.0, 5.5)	13 Fr–21 Fr Arterial
Impella RP	22 Fr Venous
TandemHeart	12 Fr–19 Fr Arterial; 21 Fr Venous
VA-ECMO	14 Fr–19 Fr Arterial; 17–21 Fr Venous
TAVR	
Sapien 3 (Edwards Lifesciences)	14 Fr Arterial (20, 23, 26 mm); 16 Fr Arterial (29 mm)
Evolut R (Medtronic)	16 Fr Arterial (23, 26, 29, 34 mm)
Navitor (St. Jude Medical)	14 Fr, 15 Fr Arterial (23, 25, 27, 29 mm)
ACURATE neo2™(Boston scientific)	14 Fr Arterial (23, 25, 27 mm)
TEER	
MitraClip (Abbott)	24 Fr Venous
Triclip tricuspid valve repair (Abbott)	25 Fr Venous
Percutaneous LAAO	
Watchman (Boston scientific)	14 Fr Venous
Amplatzer amulet (Abbott)	12–14 Fr Venous
Interatrial shunts	
Amplatzer talisman occluder (Abbott)	8 Fr–9 Fr Venous
CARDIOFORM (Gore)	10 Fr Venous
Figulla Flex II (Occlutech International)	7 Fr–11 Fr Venous
NobleStitch (HeartStitch)	14 Fr Venous
CeraFlex occluder (Lifetech Scientific)	9 Fr–14 Fr Venous
NitiOcclud (PFM Medical)	9 Fr–10 Fr Venous
Ultrasept (Cardia Inc.)	10 Fr–11 Fr Venous

TAVR, transcatheter aortic valve replacement; TEER, transcatheter edge-to-edge repair; VA-ECMO, veno-arterial extracorporeal membrane oxygenation.

## Conclusion

Despite technologic advances in transcatheter-based therapies using minimally invasive techniques, major bleeding and vascular complications continue to hinder outcomes across the spectrum of contemporary interventional cardiology. While cardiovascular societies have put forth guidelines around proper management of arterial access and closure, the data supporting these recommendations have either been observational in design or derived from medium-sized clinical trials. As a result, there remains an unmet need for further research in the form of large multi-center registries with nested and pragmatic clinical trials to address the knowledge gaps that remain in this field. Clinical questions that remain unanswered and could potentially optimize catheter-based procedures include the role that short-acting parenteral antiplatelet agents may play in reducing the risk for periprocedural bleeding, the potential merits of pre-emptive atherectomy and/or lithotripsy of calcified iliofemoral vessels in patients with peripheral arterial disease who are undergoing large bore procedures, the development of smaller caliber delivery platforms to facilitate MCS and structural heart interventions, and the creation of novel bleeding detection platforms capable of recognizing hemorrhagic sequelae prior to overt clinical manifestation (130–132). Until then, concerted efforts should be made to minimize variability in clinical practice by developing institutional protocols and competency pathways incorporating best practices for safe and effective vascular access and closure. Coupled with institutional mechanisms for oversight and quality improvement, these measures hold promise in informing multicenter collaborations and clinical research designs aimed at advancing the care of patients undergoing catheter-based cardiovascular procedures.
